# A Call for the Empirical Investigation of Tear Stimuli

**DOI:** 10.3389/fpsyg.2020.00052

**Published:** 2020-01-31

**Authors:** Sarah J. Krivan, Nicole A. Thomas

**Affiliations:** ^1^Department of Psychology, Applied Attention and Perceptual Processing Laboratory, College of Healthcare Sciences, James Cook University, Cairns, QLD, Australia; ^2^Applied Attention and Perceptual Processing Laboratory, School of Psychological Sciences, Turner Institute for Brain and Mental Health, Monash University, Melbourne, VIC, Australia

**Keywords:** tear effect, face perception, adult crying, emotion, interpersonal communication, crocodile tears

## Abstract

Emotional crying is a uniquely human behavior, which typically elicits helping and empathic responses from observers. However, tears can also be used to deceive. “Crocodile tears” are insincere tears used to manipulate the observer and foster prosocial responses. The ability to discriminate between genuine and fabricated emotional displays is critical to social functioning. When insincere emotional displays are detected, they are most often met with backlash. Conversely, genuine displays foster prosocial responses. However, the majority of crying research conducted to date has used posed stimuli featuring artificial tears. As such it is yet to be determined how the artificial nature of these displays impacts person perception. Throughout this article, we discuss the necessity for empirical investigation of the differences (or similarities) in responses to posed and genuine tearful expressions. We will explore the recent adoption of genuine stimuli in emotion research and review the existing research using tear stimuli. We conclude by offering suggestions and considerations for future advancement of the emotional crying field through investigation of both posed and genuine tear stimuli.

## Introduction

Why do we cry? Emotional crying is a uniquely human display that has fascinated both scientists and lay people alike; this interest stems from an attempt to determine the functions of adult emotional tearing. A popular theory is that emotional tears serve a communicative function (Hendriks et al., 2008; Reed et al., 2015; Vingerhoets et al., 2016). Although tears have been readily touted as an honest signal of emotion (Trimble, 2012; Vingerhoets, 2013), there is a lack of empirical evidence to justify this perception. Furthermore, tears reliably elicit empathic responses (Lockwood et al., 2013) and social support from observers (Vingerhoets et al., 2016), while also signaling appeasement, which serves to reduce aggression (Hasson, 2009). While the presence of tears on a face can signal the need for social support, tears are also used to manipulate and deceive.

The accurate detection of emotional deception is critical to social functioning. Fake tears or “crocodile tears” are an insincere tearing display that evokers use to elicit sympathy and support from observers. How evokers produce these insincere tears is not yet known. Crocodile tears are typically associated with the disingenuous tears of celebrities and politicians (Manusov and Harvey, 2011), and conveying fabricated remorse during criminal court proceedings (ten Brinke et al., 2012). Alexander (2003) found that insincere narcissistic crying appears empty and orchestrated, and that witnessing this tearful display results in feeling uneasy and unmoved in a therapeutic environment. As such, insincere emotional displays elicit negative responses (Hideg and van Kleef, 2017) and reduced trust (Krumhuber et al., 2007). However, crocodile tears could also be elicited *via* deep acting where the evoker draws on previous experience in an effort to feel the emotion they are displaying (Lu et al., 2019). As such, tears elicited in this manner are driven by genuine feeling, however, are acted and physically effortful by nature. Given that tears are known to increase perceptions of remorse during apologies (Hornsey et al., 2019), and remorse is an important factor in sentencing and parole hearings (Bandes, 2016), further research exploring how we distinguish between sincere and crocodile tears is needed.

Despite the negative connotations associated with insincere emotion, most crying research has used standardized or posed faces featuring artificial tears. Although these studies have demonstrated that tears are responded to favorably (Balsters et al., 2013; Lockwood et al., 2013), how the artificial nature of these displays impacts person perception is yet to be determined. We call for the empirical investigation of the perception of both posed and genuine emotional tear displays. We first discuss the movement toward the adoption of genuine and ecologically valid stimuli in emotion research. Then, we explore research utilizing images of crying faces and highlight the advancements achieved and potential implications for the posed face methodology. Furthermore, we discuss recommendations for future research that highlight the perceptual differences between posed and genuine tearful displays. Finally, we conclude it is necessary to explore perceptions of genuine and disingenuous crying and believe posed and genuine stimuli can aid in this investigation.

## Genuine Emotional Displays

The recent movement toward using genuine expressions has predominantly stemmed from human ability to determine the genuineness of emotional displays (McLellan et al., 2010). Primarily, genuine expression research has investigated the difference between Duchenne and non-Duchenne smiles (Duchenne, 1862/1990; Ekman et al., 1990). Smiles are characterized by the activation of the zygomaticus major (i.e., the muscle responsible for drawing the corners of the mouth upward), while Duchenne smiles feature both zygomaticus and orbicularis oculi activation (i.e., the muscle associated with the crinkling of the eyes). Duchenne smiles are reliably judged as more intensely happy (Leppänen and Hietanen, 2007), and are mimicked more than non-Duchenne smiles (Krumhuber et al., 2014). Additionally, when mimicry is constrained, people are less accurate at recognizing emotional expressions (Oberman et al., 2007) and they display a reduced ability to discriminate between posed and genuine smiles (Rychlowska et al., 2014).

Compared to happiness, the literature exploring genuine displays of sadness is limited. Despite this reduced inquiry, findings are similar to smiling research. McLellan et al. (2010) confirmed that participants can discriminate between posed and genuine sadness equally as well as happiness. In a follow-up study, genuine happy and sad displays resulted in greater neural activation in brain regions associated with emotion recognition relative to posed expressions (McLellan et al., 2012). Applied research by Hackett et al. (2008) revealed that participants who expected rape victims to be emotionally expressive perceived crying victims to be more credible than non-criers. Given that Hornsey et al. (2019) found tearful apologies were more remorseful, it seems that viewer expectations about tears in negative displays are particularly important. Moving forward, research will need to encompass a wider variety of tearing stimuli to afford an understanding of how insincere crocodile tears are distinguished from genuine emotion.

Caveats associated with the use of genuine emotional stimuli stem from the time-consuming, labor-intensive demands of creating these displays, as well as less experimental control. For these reasons, some researchers have employed blended emotional displays where smiles are paired with eye-displays that feature expressions other than happiness (Gutiérrez-García and Calvo, 2015). Although this research has offered useful information about facial markers, these expressions are not authentic. As such, future investigations should explore whether people rely on facial markers to determine authenticity, or if they discriminate between shown and felt emotions. An interesting alternative to the caveats associated with the generation of genuine stimuli stems from a normative study by Dawel et al. (2017). While several posed facial databases, most notably the Pictures of Facial Affect database (Ekman and Friesen, 1976), were not perceived as showing genuine emotion, other posed facial expressions were perceived as genuine. Thus, posed perceived-as-genuine expressions offer a compromise to the difficulties associated with generating authentic stimuli, while allowing additional control. This advancement is particularly important for tear research, as it is currently unknown whether posed-tearful displays are perceived as perceptually genuine. As such, it is important that future investigations explore whether there are differences (or similarities) between the posed expressions typically used in crying research and genuine tearful stimuli.

## The Artificial Tear

Most existing research investigating the perception of emotional tearing uses posed facial expressions that feature artificial tears, added using eyedrops or digital enhancement (Reed et al., 2015; Ito et al., 2019). These artificial images have been used to explore how the presence of tears influences the perception of sadness (Hendriks et al., 2007; Ito et al., 2019), and the degree of helping behaviors elicited (Hendriks and Vingerhoets, 2006; Balsters et al., 2013; Lockwood et al., 2013). When images with visible tears are perceived as significantly sadder than the same image without tears, it is referred to as the *tear effect* (Provine et al., 2009).

In exploring perceptions of sadness, tears are typically added to sad and neutral faces, and various measures (e.g., reaction time, rating scales, and electroencephalography) are employed to examine how tears are perceived (Hendriks et al., 2007; Balsters et al., 2013). Balsters et al. (2013) demonstrated that even when brief presentations of tearful sad and neutral faces are shown, participants correctly categorize perceived sadness faster for sad expressions with tears, relative to sad and neutral tear-free expressions. However, contradictory evidence has been demonstrated when exploring the affective ratings of Duchenne smiles featuring tears. Reed et al. (2015) demonstrated that a tearful Duchenne smile was perceived as more intense than the tear-free counterpart. Furthermore, a trend toward increased happiness ratings was observed for the tearful Duchenne face. Thus, it is possible that Duchenne smiles signify genuine joy when they are accompanied by tears, akin to a dimorphous expression. Research exploring dimorphous event-elicited expressions of tearful-joy has identified that context is essential to the perception of tearful-joy as positive; as in the absence of context, the emotions were perceived as negative (Aragón, 2017). Thus, further work investigating whether posed happy-tear displays and genuine happy-tear displays are perceptually distinct is a worthy avenue of future research.

Recently, researchers have investigated whether the *tear effect* extends beyond sad, happy, and neutral expressions, as tears are elicited in response to a variety of emotions (Vingerhoets, 2013). Ito et al. (2019) concluded that the presence of tears on all negative emotions rendered them more perceptually similar to sadness, when examined in multidimensional space. Reed et al. (2015) further explored the *tear effect* using dynamic prototypical displays of anger, fear, disgust, sadness, and neutral expressions. An actress posed these expressions twice, once as traditional expressions, and once after using eyedrops to simulate tears. Importantly, no differences in the perceived authenticity of the displays were observed between tearful and non-tearful expressions. When examining intensity, valence, and emotion-specific ratings, further generalized support was demonstrated for the *tear effect* and the role of tears as a marker of sadness. Although Reed et al. (2015) found no perceptual differences in authenticity between tearful and non-tearful expressions, no other study has considered the influence of perceived genuineness. However, people are able to distinguish between posed and genuine sadness (McLellan et al., 2010). Thus, further research is needed to determine whether people are able to distinguish between posed and genuine tearful displays.

In the context of our everyday lives, it is of interest to understand the relationship between tears, emotional support, and empathy. There is consensus that tears elicit greater emotional support and empathy compared to tear-free expressions (Hendriks and Vingerhoets, 2006; Balsters et al., 2013; Lockwood et al., 2013). Hendriks and Vingerhoets (2006) concluded that tearful expressions elicit greater support and reduced avoidance behaviors relative to other emotional displays. Furthermore, tears elicited greater perceived personal distress. Thus, despite participants’ belief that encountering a tearful person would increase their own distress, they still reported greater helping responses to tears. Lockwood et al. (2013) further explored the role of empathy in response to emotional crying using reaction time. Participants responded to neutral and caregiving words after witnessing subliminally presented emotional face primes of happy, sad, and crying faces. Individuals high in cognitive empathy showed no differences in response time. However, individuals low in cognitive empathy were slower to respond to caregiving words after being primed with a crying face, but not after sad or happy expressions. Thus, the level of empathy experienced by the observer also influences how individuals respond to crying persons. Collectively, these studies demonstrate that posed facial expressions elicit empathic responses; however, they neglect to explore the role of empathy in responding to genuine versus posed displays.

To adopt more ecologically valid crying stimuli, researchers have used crying photographs from the image-sharing site Flickr. Selecting crying photographs allows for the investigation of the *tear effect* using the inverse of the artificial tear addition technique. Provine et al. (2009) were the first to demonstrate the *tear effect* using Flickr images that included tears, which were digitally removed to create tear-free duplicates. Takahashi et al. (2015) also used the tear removal paradigm in an fMRI study investigating the perception of tears on sad and neutral expressions. The *tear effect* for sad expressions featuring tears was replicated, and they further concluded that the *tear effect* was larger for neutral faces than sad faces. As tears serve as a salient marker of sadness, their presence resolved the ambiguity of the neutral faces.

Although these studies used stimuli with greater ecological validity, it is impossible to tell whether their images were perceived as authentic expressions of emotion by the participants. As Flickr is a website where people primarily upload their own images to share with friends and followers, images shared to the platform are likely posed and self-selected by the individuals to present themselves in a positive manner (Angus and Thelwall, 2010; Malinen, 2010). Thus, posed datasets allowed for the investigation of the perception of tears with rigorous control (Balsters et al., 2013; Lockwood et al., 2013), and stimuli with greater ecological validity have replicated these effects (Provine et al., 2009; Takahashi et al., 2015); however, the need for research using genuine tearful expressions remains.

## The Genuine Tear

Recently, researchers have begun to use photographic stimuli featuring emotional tearing, which were captured in a moment of genuine emotional experience. These images were captured during the Museum of Modern Art, Artist is Present exhibit, where nearly 1,000 people sat with Marina Abramović and cried during the experience. As these tears were elicited in a moment of genuine emotion, these studies have investigated the perceived warmth and competence of the crying persons (van de Ven et al., 2017; Zickfeld et al., 2018; Zickfeld and Schubert, 2018), as well as the perceived social-connectedness and willingness to provide help to crying persons (Vingerhoets et al., 2016; Stadel et al., 2019). The original study by van de Ven et al. (2017) concluded that tearful individuals were perceived as warmer, though less competent, than tear-free individuals. Two replications of this study also determined that tearful individuals were perceived as warmer; however, neither study replicated the reduced competence effect when using a larger sample of target crying faces (Zickfeld et al., 2018; Zickfeld and Schubert, 2018). Zickfeld et al. (2018) concluded that the competence effect from the original study was likely target specific, and thus the presence of tears is unlikely to alter perceptions of competence.

Importantly, the work conducted using genuine tear stimuli has also replicated the findings that emotional tears elicit support and willingness to help. Vingerhoets et al. (2016) concluded that participants attribute greater helping behaviors to individuals with tears, than without tears. Furthermore, through mediation analysis it was determined that helping behaviors stemmed from a perception of closeness to the individuals in the crying images. Similarly, tearful stimuli facilitate approach behaviors relative to avoidance (Riem et al., 2017; Gračanin et al., 2018). Furthermore, Stadel et al. (2019) identified that participants show increased willingness to help individuals with tears, and concluded that this willingness was the strongest between female and mixed dyads, compared to male dyads. Therefore, it seems that tears are a signal that elicits helping responses from observers; however, both the gender of the participant and the expressor might mediate the degree of assistance offered. Future research should expand upon these findings, which stem from self-report willingness to help measures, to better encompass whether perception is aligned with actual helping behavior. Additionally, while these stimuli were captured during a moment of genuine experience, it is unknown what the individuals were feeling. Aragón and Clark (2018) explored responses to genuine dimorphous happy tears. Participants reported a greater likelihood of down-regulation responses to tearful-joy, than joy expressed with smiles. Thus, future research needs to consider the role that emotional state plays in establishing the way that we respond to tears.

## Discussion

To date, research using images of teary expressions has focused on expressions of sadness and the anticipated perception and response of individuals. Although crying research has recently adopted the use of genuine expressions, there is no empirical evidence exploring differences in perceived authenticity between posed and genuine displays of emotion featuring tears. Table 1 provides a collation of the studies examining the tear effect, and the influence that tears have on empathic responses. This table highlights the type of stimuli used in each experiment, the method of tear addition or removal, and the effect sizes reported in the published literature. It must be noted that the type of task, the number of identities used, and the gender of the stimuli varied widely across these studies. This variability further highlights the need for empirical studies using both posed and genuine tearful expressions. This empirical investigation will assist with better understanding the perceptual differences between tear stimuli and aid in our understanding of how we discriminate genuine and posed emotion.

**Table 1:** A comparison of the effect sizes reported in published studies examining tears.

Authors	Stimulus type	Tear method	Effect size
**Faster reaction time to tearful images**			
Balsters et al. (2013)	KDEF	Digitally added	$\eta^{2}$ = 0.284^†^
Gračanin et al. (2018)	MoMA	Digitally removed	$\eta_{p}^{2}$ = 0.26^†^
Riem et al. (2017)	MoMA	Digitally removed	$\eta_{p}^{2}$ = 0.69^†^
**Greater perceived sadness for tearful images**			
Provine et al. (2009)	Flickr tear images	Digitally removed	$\eta^{2}$= 0.26^†^
Takahashi et al. (2015)	Flickr tear images	Digitally removed	$\eta_{p}^{2}$ = 0.793^*^
Reed et al. (2015)	Female actress using FACS	Eyedrops	*d* = 0.22
Ito et al. (2019)	TFEID	Digitally added	$\eta_{p}^{2}$ = 0.073
van de Ven et al. (2017)	MoMA	Digitally removed	$\eta_{p}^{2}$ = 0.15^†^
Zickfeld et al. (2018)	MoMA	Digitally removed	*d* = 0.86
**Greater willingness to help/greater perceived support for tearful images**			
Balsters et al. (2013)	KDEF	Digitally added	$\eta^{2}$ = 0.375^†^
Vingerhoets et al. (2016)	MoMA	Digitally removed	*d* = 0.85–1.32
Zickfeld and Schubert (2018)	MoMA	Digitally removed	*d_S_* = 0.70–0.82

*KDEF, Karolinska Directed Emotional Faces; MoMA, Genuine tear expressions captured during Museum of Modern Art Performance; TFEID, Taiwanese Facial Expression Image Database; Flickr tear images, images of tearful individuals found on Flickr (unknown if genuine or posed). Effect sizes are reported as in the published papers*.

* *Denotes that original paper did not report effect size, and thus it was estimated from main effect of tears*;

† *Denotes effect size from main effect*.

Furthermore, as the majority of the work conducted to date has used posed expressions, there has been limited focus on the other facial responses that accompany emotional tears, including blotchy faces and bloodshot eyes (Provine et al., 2011, 2013). Küster (2018) explored the influence of tears and pupil size on the perception of sadness using digital avatars. While both the presence of tears and smaller pupil sizes increased perceived sadness, there was no interaction effect between tears and pupil size. The inverse consideration of the extreme features accompanying emotional crying is the perceptual and affective differences between tearing up and crying uncontrollably (i.e., ugly crying). Research using vignettes has demonstrated that the intensity of tears moderates observer reactions, where in some scenarios just tearing up may elicit more positive responses than weeping (Wong et al., 2011). Thus, further work in this field should explore the relationship between the intensity of the tears and observer responses. It may be that assistance for emotional crying is curvilinear, where there is an optimum level of tearing that elicits helping responses from observers.

Finally, the adoption of investigative techniques like psychophysiology may offer insight into the perceptions of tears to further corroborate the results from self-report studies. Recently, mirror neurons have been proposed as a mechanism for sharing others’ emotional states, with “feeling” and “perceiving” emotion sharing neural substrates (Wicker et al., 2003; Singer et al., 2004). Similarly, facial mimicry studies have identified that when participants’ ability to mimic is impaired, they show reduced emotion recognition abilities (Oberman et al., 2007; Rychlowska et al., 2014). In addition, examination of other physiological techniques, such as eye-tracking and galvanic skin response, may yield fruitful information about the features that individuals attend to in decoding an emotional face, and the degree of arousal that tearful expressions elicit. Analysis of the arousal response may assist in determining the motivation for the helping behaviors as a metric of personal distress. Furthermore, the inclusion of psychophysiological metrics allows for greater certainty in the true nature of the self-report responses.

In this paper, we have reviewed recent work using facial expressions as a means of investigating inter-individual functions of crying. Reviewing these studies has revealed that the use of posed expressions has afforded an understanding of the communicative functions of emotional tears by employing rigorously controlled stimuli between conditions. In addition, the use of genuine expressions of emotion in more recent crying research has replicated findings that both posed and genuine expressions of emotion are effective at eliciting support and attention. However, whether posed tearful expressions are being treated as perceptually authentic, or if their staged nature is impacting upon person perception is yet to be determined. Thus, to continue advancing the understanding about the interpersonal functions of human emotional tearing, we need to adopt an approach that better explores how we perceive both genuine and non-genuine crying expressions. This advancement needs to encompass a greater range of tearing stimuli to allow for the exploration of the physiological effects that accompany emotional tearing. This research will provide a basis for understanding the type of emotional tears we respond to. People are able to distinguish between posed and genuine emotions, yet tears have not received this same inquiry. Determining how we distinguish between posed and genuine tearful expressions will aid in further understanding the functions of this uniquely human phenomenon.

## Author Contributions

SK conceptualized and designed the article and wrote the first draft of the manuscript. SK and NT contributed to manuscript revision, read and approved the submitted version.

### Conflict of Interest

The authors declare that the research was conducted in the absence of any commercial or financial relationships that could be construed as a potential conflict of interest.
